# The selection of indicators from initial blood routine test results to improve the accuracy of early prediction of COVID-19 severity

**DOI:** 10.1371/journal.pone.0253329

**Published:** 2021-06-15

**Authors:** Jiaqing Luo, Lingyun Zhou, Yunyu Feng, Bo Li, Shujin Guo

**Affiliations:** 1 School of Computer Science and Engineering, University of Electronic Science and Technology of China, Chengdu, China; 2 Center of Infectious Diseases, West China Hospital of Sichuan University, Chengdu, China; 3 State Key Laboratory of Biotherapy and Cancer Center, West China Hospital, Sichuan University and Collaborative Innovation Center, Chengdu, China; 4 Department of Otorhinolaryngology, Head & Neck Surgery, West China Hospital, Sichuan University, Chengdu, China; 5 The Geriatric Respiratory Department, Sichuan Provincial People’s Hospital, University of Electronic Science and Technology of China, Chengdu, China; University of Defence in Belgrade, SERBIA

## Abstract

The global pandemic of COVID-19 poses a huge threat to the health and lives of people all over the world, and brings unprecedented pressure to the medical system. We need to establish a practical method to improve the efficiency of treatment and optimize the allocation of medical resources. Due to the influx of a large number of patients into the hospital and the running of medical resources, blood routine test became the only possible check while COVID-19 patients first go to a fever clinic in a community hospital. This study aims to establish an efficient method to identify key indicators from initial blood routine test results for COVID-19 severity prediction. We determined that age is a key indicator for severity predicting of COVID-19, with an accuracy of 0.77 and an AUC of 0.92. In order to improve the accuracy of prediction, we proposed a Multi Criteria Decision Making (MCDM) algorithm, which combines the Technique for Order of Preference by Similarity to Ideal Solution (TOPSIS) and Naïve Bayes (NB) classifier, to further select effective indicators from patients’ initial blood test results. The MCDM algorithm selected 3 dominant feature subsets: {Age, WBC, LYMC, NEUT} with a selection rate of 44%, {Age, NEUT, LYMC} with a selection rate of 38%, and {Age, WBC, LYMC} with a selection rate of 9%. Using these feature subsets, the optimized prediction model could achieve an accuracy of 0.82 and an AUC of 0.93. These results indicated that Age, WBC, LYMC, NEUT were the key factors for COVID-19 severity prediction. Using age and the indicators selected by the MCDM algorithm from initial blood routine test results can effectively predict the severity of COVID-19. Our research could not only help medical workers identify patients with severe COVID-19 at an early stage, but also help doctors understand the pathogenesis of COVID-19 through key indicators.

## Introduction

Currently, more than 40 million people worldwide are infected with the SARS-Cov-2 virus, and more than 10 million people are suffering from Coronavirus disease 2019 (COVID-19) and are receiving treatments [1]. This poses a huge threat to the health and lives of people all over the world, and brings unprecedented pressure to the medical system. Many infected patients cannot receive timely and effective treatment, and it will also reduce the treatment efficiency of other emergency patients [2].

Patients with suspicious symptoms and epidemiological history first visit the fever clinic of the community hospital [3]. They usually undergo three initial tests: SARS-Cov-2 RNA confirms SARS-Cov-2 infection, blood routine test, and chest CT scan to initially assess the severity of COVID-19 [4]. The timely and effective triage of COVID-19 patients based on the results of the three initial tests is of great significance for maintaining emergency capacity and optimizing treatment plans [2].

Although most COVID-19 patients are Mild-Moderate cases and can recover on their own, about 14% of patients are Severe cases, and 5% of patients are Critically Severe cases [5]. Severe-Critically Severe cases usually develop Acute Respiratory Distress Syndrome (ARDS) or Multiple Organ Dysfunction Syndrome (MODS) within two weeks of infection [6], which consumes most of the medical resources and leads to a high case fatality rate (up to 49%) [5, 6]. Early prediction of the severity of COVID-19 can help quickly triage patients (i.e., quarantine, hospital admission or ICU assignment, etc.) and optimize the use of medical resources and timely medical intervention [7, 8]. Blood routine test is the most basic examination. The blood routine test results include red blood cell count (RBC), hemoglobin (HGB), platelets (PLT), white blood cell count (WBC), lymphocyte count (LYMC), lymphocyte ratio (LYMPH), neutrophil count (NEUT), neutrophil ratio (NEU) neutrophil to lymphocyte ratio (NLR), etc. [9–11]. For infectious diseases, a substantial increase or decrease of WBC prompt the severity of the infection. The number and proportion of NEUT can be used to determine the presence or absence of bacterial infection. The rise or fall of LYMC is a characteristic of viral infection [12]. Decreasing of lymphocytes is one of the most critical features of SARS-Cov-2 infection [13]. Of all the initial tests for COVID-19 patients, blood routine test is the worldwide common test with good consistency, and the results are usually available within 2 hours. Due to the influx of a large number of patients into the hospital and the running of medical resources, blood routine test might be the only possible check while COVID-19 patients first go to a fever clinic in a community hospital [4].

When an emerging infectious disease breaks out, we need to quickly understand its pathogenic characteristics and independent risk factors that affect its progression [14]. At this time, the outbreak area is often limited, and the number of patients is small at the very beginning [3]. How to comprehensively analyze the high-risk factors leading to severe illness in a small sample is a serious clinical challenge [15]. Up to now, there have been many studies on predicting the severity of the COVID-19 (i.e., older age, pulmonary micro-thrombosis, increased inflammatory factors (C-reactive protein (CRP), IL-6), hyper-lactic acidemia, D-dimer progressive heightened, decreased lymphocyte count (especially CD8+ T cell count) and short-term progression of lung lesions, etc.) [7, 16–19]. However, the collection of these indicators requires multiple tests and takes a lot of time [19]. These studies certainly can help us improve the treatment, but can hardly help us quickly respond to emerging infectious disease outbreaks [20–22].

In this paper, we aimed to select features from initial blood test results to predict the severity of COVID-19 quickly and accurately. We first defined feature selection as a Multiple Criteria Decision Making (MCDM) problem that considers the correlation between input features and the correlation between input and output features [23–26]. In MCDM, some methods provide the priority of indicators, while others provide the ranking of indicators. One of the MCDM ranking methods is the Technique for Order of Preference by Similarity to Ideal Solution (TOPSIS), which has been used in the selection of significant risk factors for healthcare and prognosis [27–29]. Different from the existing TOPSIS methods [8, 27], we use maximum relevance and minimum redundancy [30–32] as the criteria for feature selection in order to select independent risk factors. The maximal relevance feature is to select the input features with the highest relevance to output features. The combinations of individually good features do not necessarily lead to good classification performance [30, 31]. The minimal redundancy is to reduce the redundancy among input features. We then used a series of intuitive measures of relevance and redundancy to select independent risk factors. Finally, we use Naïve Bayes (NB) classifier to achieve the highest prediction accuracy with the fewest input features. Using TOPSIS MCDM, we successfully screened out "independent risk factors" that predict the severity of COVID-19 [25].

Our research established an easy and accurate method for early predict the severity of COVID-19 based on the simple clinic characteristics, which could help medical workers identify patients with severe COVID-19 at an early stage, improve the efficiency of emergency triage of patients, and help doctors understand the pathogenesis of COVID-19 through key indicators.

## Methods

### Patient enrollment and study design

We performed this prospective cohort study from March 15 to March 20, 2020 in Wuhan Red Cross Hospital, a hospital designated to treat COVID-19 in Wuhan, China. We collected 196 COVID-19 patients diagnosed according to WHO guidance [33] from February 1, 2020 to March 15, 2020. The inclusion criteria were as follows: (1) diagnosis of COVID-19 pneumonia according to the WHO interim guidance published on 28 January 2020 (ref), and (2) availability of relevant medical record information, especially initial blood test results when patients first go to a fever clinic in a community hospital and patients’ severity. Patients discharged within 24 h since admission were excluded.

### Ethics

The study was approved by the ethics committee of Sichuan Provincial People’s Hospital. Since it is not allowed to take any paper documents out of the quarantine area of Wuhan Red Cross Hospital, all participants have obtained oral informed consent, which is recorded by the doctor and kept in the medical record. Before building the predictive model, all data was completely anonymized and cleared.

### Definitions

COVID-19 was confirmed by detecting SARS-CoV-2 RNA test. According to the 5th edition of the China Guidelines for the Diagnosis and Treatment Plan of COVID-19 Infection by the National Health Commission (Trial Version 5) [34], the cases were classified into Mild-Moderate and Severe- Critically Severe.

### Data collection

The following information was extracted from each patient: Gender, Age and patients’ initial blood routine test results including WBC, LYMC, LYMPH, NEUT, NEU and NLR. The dataset contained 8 input features {Gender, Age, WBC, LYMC, LYMPH, NEUT, NEU, NLR}, and 1 output feature (Severity).

### Statistical analysis

Quantitative variables were expressed as the mean ± standard deviation or the median with interquartile ranges, while categorical variables were expressed as absolute and relative frequencies. The t test or Wilcoxon-test was performed to calculate differences between quantitative data; and χ2 test was performed to calculate differences between qualitative data. According to the data characteristics, the correlation between clinic characteristics and COVID-19 severity was calculated according to Kendall correlation coefficient (Gender-severity) or Spearman correlation coefficient. Logistic regression analysis was performed for independent variables with collinearity. Wald test was used to determine the joint significance of variables. The standard deviation was used to measure dispersion degrees. Statistical procedures were performed with R statistical software. P values of ≤0.05 were considered significant.

### The MCDM algorithm design and implementation

The proposed algorithm is basically designed for predicting COVID-19 severity, either Mild-Moderate or Severe-Critically Severe case. It reduces computation time, improves prediction performance, and a better understanding of the data in machine learning. It consists of 4 major stages: preprocessing, feature ranking, feature selection and performance evaluation. Preprocessing is the process to refine the collected raw data to de-noise it. Feature ranking is the process of ordering the features by the value of some scoring function, which usually measures feature-relevance. Feature selection aims to choosing a small subset of the relevant features from the original features by removing irrelevant, redundant, or noisy features. Performance evaluation is to measure the performance of the binary classification by statistical measures, i.e., Accuracy (ACC), True Positive Rate (TPR), False Positive Rate (FPR) and F1 score.

#### Preprocessing

We use stratified random sampling to divide the dataset into 2 subsets: training set (80%) and test set (20%). In these 4 stages, we only used the test set for performance evaluation. Suppose there are m input features and n output features. Let *X* = {x|1≤x≤m} be the input feature set and *Y* = {y|m+1≤y≤m+n} be the output feature set. Elements x and y are indexes of features. The feature set is F = *X*∪*Y* = {i|1≤i≤m+n}. We calculated and visualized a (m+n)×(m+n) correlation matrix R and a (m+n)×(m+n) p-value matrix P to show the correlations between all different feature pairs. To simplify the analysis, we then preprocess R in 2 steps. STEP1: We ignored the sign of R[i,j]. Let R[i,j] = |R[i,j]| so that the range of R[i,j] changes from [–1,1] to [0,1], where i, j∈*F*. STEP2: We filtered R through P. For x∈*X* and y∈*Y*, if P[x,y] = P[y,x] > 0.05, R[x,y] and R[y,x] are not significant. We set R[x,y] = R[y,x] = 0 and R[x,i] = R[i,x] = 1 for i∈*X*.

#### Feature ranking

We defined a labeled feature set L and initialized with *L* = ∅. We iterated the procedure of ranking input features x∈*X* and moved the first in each ranking from X to L. The ranking criteria includes 2 evaluations: EVAL1: The correlation between input feature x∈*X* and output feature y∈*Y*, R[x,y] or R[y,x]. EVAL2: The correlation between input feature x∈*X* and labeled feature v∈*L*, R[x,v] or R[v,x]. This explicitly evaluates multiple conflicting criteria in decision making. We proposed an algorithm to solve this Multiple Criteria Decision Making (MCDM) problem by using the Technique for Order of Preference by Similarity to Ideal Solution (TOPSIS), which is a compensatory aggregation method. The algorithm, called MCDM, creates an evaluation matrix E consisting of p criteria and q alternatives, to rank input features. According to Pareto’s principle, the algorithm divided x into the following 2 types:

TYPE1: If |*X*|>min {m−1, ⌈0.8×m⌉}, x to be labeled are core features (the top 20%), which should have the lowest R[v,x] from EVAL2, and the highest R[y,x] from EVAL1. The algorithm sorts the elements of sets *L*∪*Y* and X in ascending order to get sequences ${\left(r_{i}\right)}_{i=1}^{|L|+n}$ and ${\left(c_{j}\right)}_{j=1}^{\left|X\right|}$, respectively. Let p = |*L*|+n and *q* = |X|, the algorithm extracts a p×q submatrix E from R such that *E*[i, j] = *R*[*r*_*i*_, *c*_*j*_]. The worst condition of *E*[i, j] is $w_{i}=\left\{ \begin{array}{c} 1,\;i\leq |L|\\ 0,\;i>|L| \end{array}\right.$, and the best condition of *E*[i, j] is $b_{i}=\left\{ \begin{array}{c} 0,\;i\leq |L|\\ 1,\;i>|L| \end{array}\right.$.
TYPE2: If |*X*|≤min {|*m*−1, ⌈0.8×m⌉}, x to be labeled are auxiliary features (the rest 80%), which only need to have the lowest R[v,x] from EVAL2. The algorithm sorts the elements of sets *L* and X in ascending order to get sequences ${\left(r_{i}\right)}_{i=1}^{\left|L\right|}$ and ${\left(c_{j}\right)}_{j=1}^{\left|X\right|}$, respectively. Let p = |*L*| and q = |*X*|, E is a p×q matrix with *E*[i, j] = *R*[*r*_*i*_, *c*_*j*_].

The algorithm calculates the L2-distance between the target alternative j and the worst condition:

$$d_{wj}=\sqrt{\sum_{i=1}^{p}{\left(E[i,j]-w_{i}\right)}^{2}}$$ Eq 1

It then calculates the distance between j’s condition and the best condition:

$$d_{bj}=\sqrt{\sum_{i=1}^{p}{\left(E[i,j]-b_{i}\right)}^{2}}$$ Eq 2

After that, it calculates the similarity to the worst condition:

$$s_{j}=\frac{d_{wj}}{d_{wj}+d_{bj}},\;0\leq s_{j}\leq 1$$ Eq 3

*s*_*j*_ = 1 if and only if alternative j has the best condition, and *s*_*j*_ = 0 if and only if alternative j has the worst condition. Let $j^{*}=\underset{j}{\arg\;\max}\left\{s_{j}\right\}$, then *X* = *X*\{*c*_j*_} and *L* = *L*∪{*c*_j*_}.

The pseudocode of the MCDM algorithm is as follows:

Algorithm MCDM is

Input: correlation matrix R, number of input features m, number of input features n, input feature set X, output feature set Y

Output: labeled feature set L

initialize *L* = ∅

while *X* ≠ ∅ do

    if |*X*|>min {m−1, ⌈0.8×m⌉}

        ${\left(r_{i}\right)}_{i=1}^{|L|+n}$←sort *L*∪*Y* and X in ascending order

    else

        ${\left(r_{i}\right)}_{i=1}^{\left|L\right|}$←sort L in ascending order

    ${\left(c_{j}\right)}_{j=1}^{\left|X\right|}$←sort X in ascending order

    extract E from R such that *E*[i, j]←*R*[*r*_*i*_, *c*_*j*_]

    for j = 1 to q do // q is the number of columns of E

        *d*_*wj*_ ←Eq 2

        *d*_*bj*_ ←Eq 3

        *s*_*j*_← Eq 4

    j*←$\underset{j}{\arg\;\max}\left\{s_{j}\right\}$

    X←*X*\{*c*_j*_}

    L← *L*∪{*c*_j*_}

    print L

return L

#### Feature selection

The goal of feature subset selection is to find the optimal input feature subset. We gradually increased the number of labeled features, and trained the model with Naïve Bayes classifier in turn. To find the optimal subset, we sequentially tested the accuracy of trained models on the training set.

#### Performance evaluation

In order to test the stability of the algorithm and observe the influence of the dataset uncertainty on feature selection, we divided the data set 100 times (80% training set and 20% test set) and repeatedly run the algorithm. We used the test set to analyze the performance of feature selection from ACC, TPR, FPR and F1 score.

### Evaluation of the predictive value of selected features

According to stratified random sampling, we divided the data set into 2 subsets: 80% of the “training set” and 20% of the “testing set”. We used Receiver Operating Characteristic (ROC) curve analysis to calculate the Area Under the Curve (AUC) and use “ROC” package in R to evaluate the prediction accuracy of our model.

## Results

### Baseline characteristics

We analyzed the data of 196 COVID-19 patients, of which 90 and 106 were male and female patients. After clearing the data set, there is no abnormal data (S1 Fig). Table 1 lists the detailed baseline characteristics. The mean age of patients was 57.74±15.87 years old. The COVID-19 patients’ initial blood routine test results showed that the WBC was 6.75±3.49◊10^9^/L; LYMC was 1.12±0.58◊10^9^/L; LYMPH was 19.91±11.52%; NEUT was 5.13±3.46◊10^9^/L; NEU was 71.34±15.24%; NLR was 7.45±13.08.

**Table 1:** Clinical features of COVID-19 cases.

Clinic Characteristics			Total Data (n = 196)	Severity of COVID-19	
			Mean (SD)	Mild-Moderate	Severe-Critically Severe
Gender	(Male/Female)		90/106	27/40	63/66
Age	(Years old)		57.74 (15.87)	45.28 (10.33)	64.21 (14.33)
Blood Routine Test Results	WBC	(1◊10^9^/L)	6.75 (3.49)	5.84(2.30)	7.23 (3.89)
	LYMC	(1◊10^9^/L)	1.12(0.58)	1.46(0.55)	0.94(0.51)
	LYMPH	(%)	19.91(11.52)	27.00 (11.07)	16.23 (9.96)
	NEUT	(1◊10^9^/L)	5.13(3.46)	3.85 (2.07)	5.79 (3.85)
	NEU	(%)	71.34(15.24)	62.27 (16.27)	76.05(12.32)
	NLR		7.45 (13.08)	3.07 (2.27)	9.72 (15.57)

### Difference in age and initial blood test results between Mild-Moderate and Severe-Critically Severe groups

**Fig 1:**
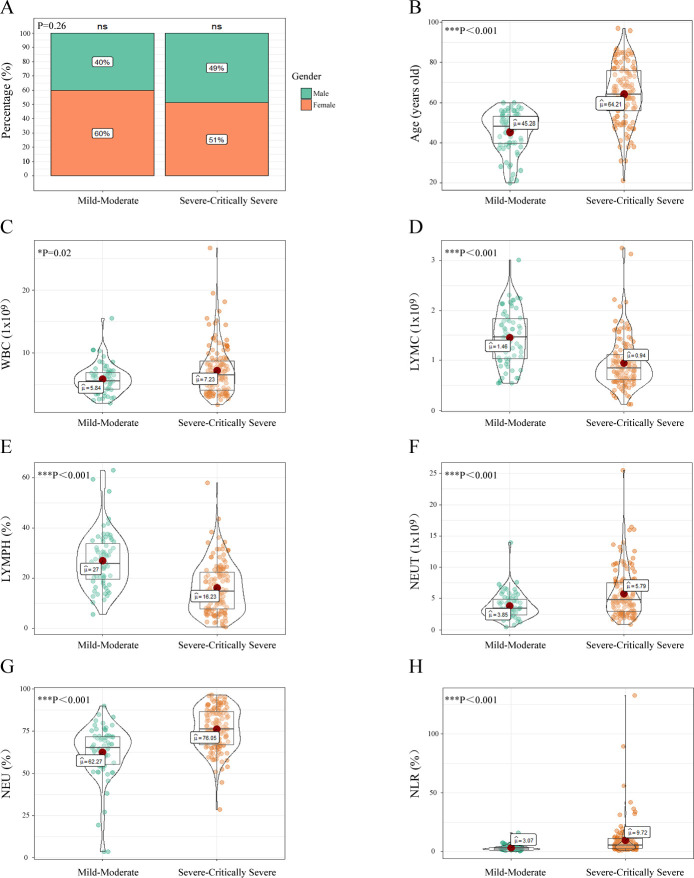
Comparison of clinic characteristics of COVID-19 patients in Mild-Moderate and Severe-Critically Severe groups (n = 196). COVID-19 were divided into Mild-Moderate and Severe-Critically Severe groups according to the 8th edition of the China Guidelines for the Diagnosis and Treatment Plan of COVID-19 Infection by the National Health Commission (Trial Version 8). (A). Gender differences between the two groups, P-value was calculated according to chi-square test. (B). Age differences between the two groups. Each plot graphically displays the central position and scatter/dispersion of the values of each group. P-value was calculated according to student t-test. (C). WBC differences between the two groups, P-value was calculated according to Wilcoxon-test. (D). LYMC differences between the two groups, P-value was calculated according to Wilcoxon-test. (E). LYMPH differences between the two groups, P-value was calculated according to Wilcoxon-test. (F). NEUT differences between the two groups, P-value was calculated according to Wilcoxon-test. (G). NEU differences between the two groups, P-value was calculated according to Wilcoxon-test. (H). NLR differences between the two groups, P-value was calculated according to Wilcoxon-test. *P <0.05, **P <0.01, ***P <0.001.

According to the 5th edition of the China Guidelines for the Diagnosis and Treatment Plan of COVID-19 Infection by the National Health Commission, we divided patients into 2 groups: 67 cases in the Mild-Moderate group, and 129 cases in the Severe-Critically Severe group (Table 1). Comparing Mild-Moderate and Severe-Critically Sever groups, the basal features showed no differences in Gender (p = 0.26) (Fig 1A). The Severe-Critically Severe group was significantly older than the Mild-Moderate group (p <0.001) (Fig 1B). The initial blood routine test seems to be important for predicting the severity of COVID-19: The Severe-Critically Severe group had a higher WBC level (p = 0.02) (Fig 1C). The Severe-Critically Severe group had extremely low LYMC (p<0.001) and LYMPH (p<0.001) (Fig 1D and 1E). In contrast, NEUT (p<0.001) and NEU (p<0.001) in the Severe-Critically Severe group were extremely high (Fig 1F and 1G). As a result, the Severe-Critically Severe group had a higher NLR (p<0.001) (Fig 1H). These observations suggest that patients’ age, and WBC, LYMC, LYMPH, NEUT, NEU, NLR from the initial blood routine test could be critical factors for predicting the severity of COVID-19.

### Predictive value of age and initial blood test results for COVID-19 severity

**Fig 2:**
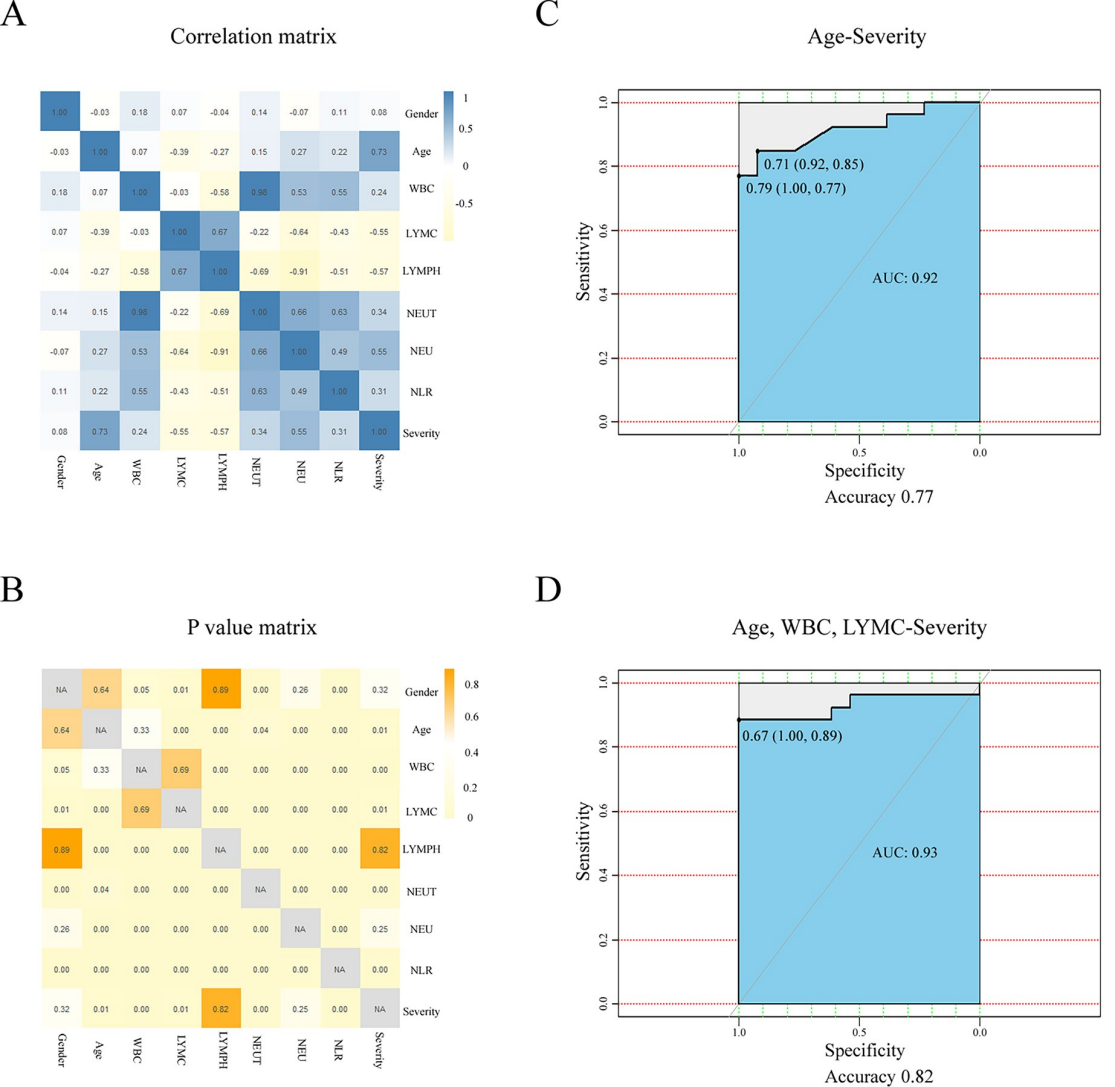
The correlation between clinic characteristics and severity of COVID-2019 and the predictive value of clinic characteristics for the severity of COVID-19. (A). Correlation analysis: Characteristics of the COVID-19 patient including Gender, Age WBC, LYMC, LYMPH, NEUT, NEU, NLR and Severity. WBC, LYMC, LYMPH, NEUT, NEU and NLR were extracted form patients’ initial blood test results. Patients were divided into Mild-Moderate and Severe-Critically Severe groups according to the 8th edition of the China Guidelines for the Diagnosis and Treatment Plan of COVID-19 Infection by the National Health Commission (Trial Version 8). According to the characteristics of the data, the correlation was calculated based on the Kendall correlation coefficient (Gender-severity) or Spearman correlation coefficient. P <0.05 was considered statistically significant. (B). P-values of correlation. (C). ROC curve used to evaluate the predictive value of Age for the severity of COVID-19 based on stratified random sampling: 80% as the training set and 20% as the testing set. (D). ROC curve used to evaluate the predictive value of Age, WBC, LYMC for the severity of COVID-19 based on stratified random sampling: 80% as the training set and 20% as the testing set.

By calculating the correlation between clinic characteristics and severity of COVID-19, we found that Age (r = 0.73, p = 0.01), WBC (r = 0.24, p<0.01), NEUT (r = 0.34, p<0.01), NLR (r = 0.31, p<0.01) were significantly positively correlated with the severity of COVID-19, while LYMC (r = -0.55, p = 0.01) was significantly negatively correlated with the severity of COVID-19 (Fig 2A and 2B). These results indicated that Age and initial blood routine test results-WBC, LYMC, NEUT, NLR, might be important for predicting the severity of COVID-19. Wald test showed that only Age was the key indicator in predicting the severity of COVID-19 (Table 2). Using stratified random sampling, we generated the ROC curve to evaluate the predictive values: 80% for the “training set” and 20% for the “testing set”. Using [18] for prediction, we can obtain an accuracy of 0.77, and an AUC of 0.92 (Fig 2C). Through dispersion analysis, we found that WBC, LYMC and LYMPH may be able to optimize prediction performance (Tables 3 and 4). The ROC curve showed that {Age, WBC, LYMC} had an accuracy of 0.82 and an AUC of 0.93 (Fig 2D). These results suggested that it is a good predictor of COVID-19 severity, but the accuracy was only 0.77. Using WBC and LYMC from initial blood routine test could rise the accuracy to 0.82.

**Table 2:** The joint significance of clinical characteristics.

Clinic Characteristics	Estimate	Std. Error	Z Value	P value	Significant
(Intercept)	-9.55	10.90	-0.88	0.38	
Gender	0.76	0.54	1.42	0.16	
Age	0.13	0.03	4.97	0.00	***
WBC	0.61	2.10	0.29	0.77	
LYMC	-0.54	2.60	-0.21	0.83	
LYMPH	-0.06	0.14	-0.42	0.67	
NEUT	-0.74	2.24	-0.33	0.74	
NEU	0.06	0.11	0.57	0.57	
NLR	0.04	0.16	0.22	0.83	

Significant

‘***’ p<0.001, ‘**’p< 0.01, ‘*’p< 0.05.

**Table 3:** Dispersion analysis of clinical characteristics.

Clinic Characteristics	Df	Deviance resid	Df	resid. Dev	P value	Significant
NULL			156	202.10		
Gender	1	0.23	155	201.87	0.63	
Age	1	63.55	154	138.32	0.00	***
WBC	1	7.20	153	131.12	0.01	**
LYMC	1	15.65	152	115.47	0.00	***
LYMPH	1	4.95	151	110.52	0.03	*
NEUT	1	0.29	150	110.23	0.59	
NEU	1	0.38	149	109.86	0.54	
NLR	1	0.06	148	109.79	0.80	

Significant

‘***’ p<0.001

‘**’p< 0.01

‘*’p< 0.05.

**Table 4:** The joint significance of different subsets.

Subset		Estimate	Std. Error	Z Value	P value	Significant
[18]	(Intercept)	-5.49	1.07	-5.10	0.00	***
	Age	0.11	0.02	5.62	0.00	***
{Age, WBC, LYMC, LYMPH}	(Intercept)	-4.43	1.80	-2.46	0.01	*
	Age	0.11	0.02	4.88	0.00	***
	WBC	0.19	0.18	1.09	0.28	
	LYMC	-1.42	0.87	-1.64	0.10	*
	LYMPH	-0.02	0.05	-0.41	0.68	
{Age, WBC, LYMC}	(Intercept)	-4.87	1.47	-3.32	0.00	***
	Age	0.11	0.02	4.89	0.00	***
	WBC	0.25	0.10	2.56	0.01	*
	LYMC	-1.73	0.45	-3.84	0.00	***

Significant

‘***’ p<0.001

‘**’p< 0.01

‘*’p< 0.05.

### Details of the MCDM algorithm to predict the severity of COVID-19

The MCDM algorithm was conducted to further investigate whether there were other factors that could improve the accuracy of prediction. The MCDM algorithm and Logistic regression analysis have obtained consistent results: Age was a key indicator in predicting the severity of COVID-19. In addition, the MCDM algorithm verified that the {Age, WBC, LYMC} subset is one of the index sets with the highest prediction accuracy.

**Fig 3:**
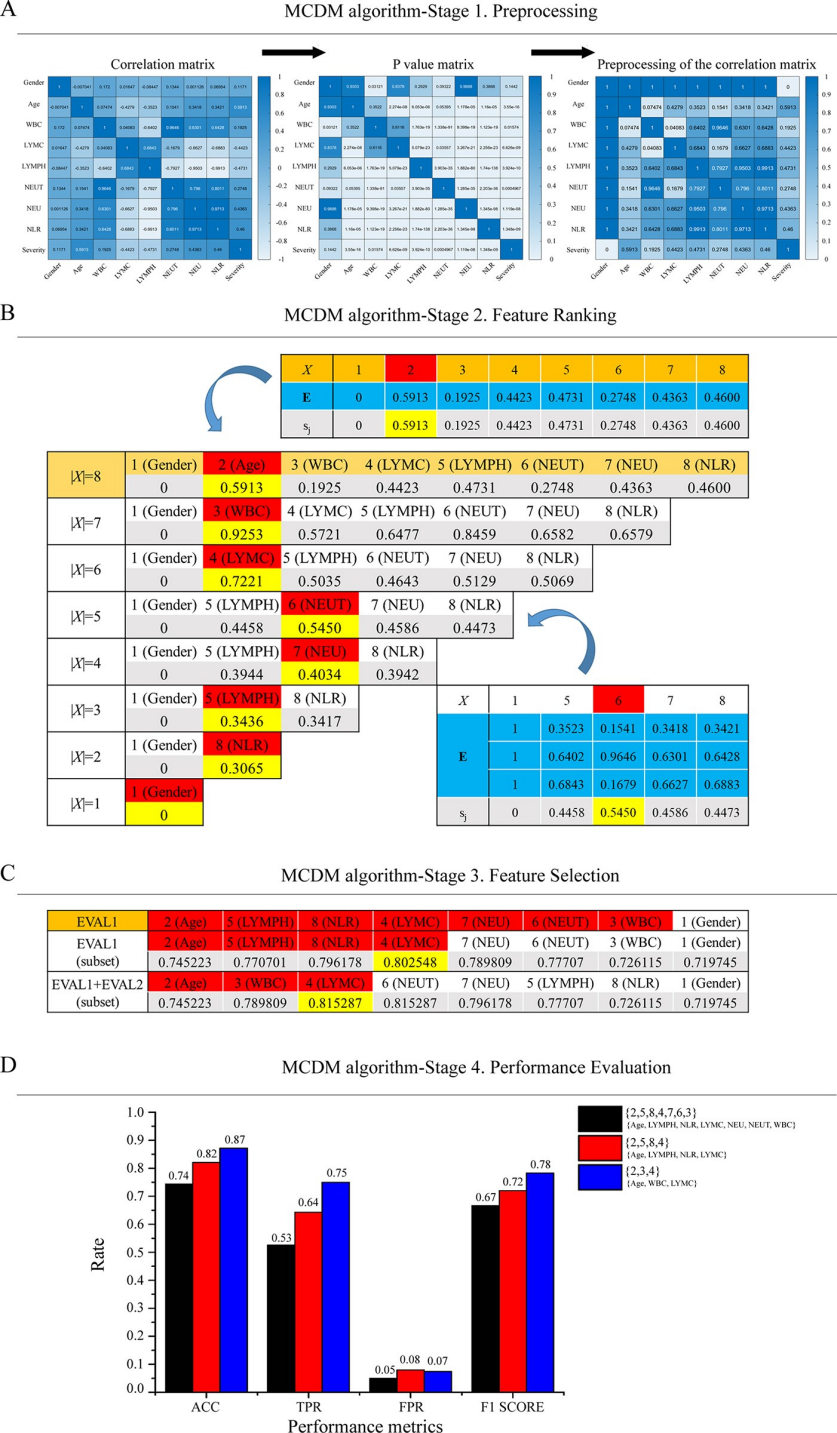
Design and implementation of the Multiple Criteria Decision Making (MCDM) algorithm for predicting the severity of COVID-19. (A). The MCDM algorithm-Stage 1. Preprocessing, this stage is the process of refining the collected raw data to eliminate noise, including correlation analysis and feature selection based on P values. Correlation was calculated according to Spearman correlation coefficient. P <0.05 was considered statistically significant. (B). The MCDM algorithm-Stage 2. Feature Ranking, this stage is the process of using the TOPSIS method to rank features. TOPSIS method: according to the severity-relevance, we defined the top 20% as the core features and the other 80% as the auxiliary feature. For key features: First, select the first feature that is most relevant to the severity; Second, select the remaining key features in turn by ranking. The ranking criteria are as relevant as possible to severity, and not relevant to the selected key features. For auxiliary features: score and rank auxiliary features according to the degree of irrelevance to key features. (C). The MCDM algorithm-Stage 3. Feature Selection, this stage is to select a subset of the features ranked by the TOPSIS method to remove irrelevant, redundant, or noisy features. EVAL1: The correlation between input features *x*∈*X* and output features y∈*Y*, *R*[*x,y*] or *R*[*y,x*]; EVAL2: The correlation between input features *x*∈*X* and labeled features v∈*L*, *R*[*x,v*] or *R*[*v,x*]; Subset: The optimal input feature subset. (D). The MCDM algorithm-Stage 4. Performance evaluation, this stage is to measure the performance of the binary classification by ACC, TPR, FPR and F1 score.

- Preprocessing (Fig 3A)—In the COVID-19 data set, m = 8 and n = 1. The 9×9 correlation matrix R, The 9×9 p-value matrix P and the range of R[i,j] for i, j∈F becomes [0,1]. Since P[1,9] = P[9,1] = 0.1442>0.05, R[1,9] and R[9,1] are not significant, R[1,9] = R[9,1] = 0, R[1,1:8] = ones(1,8) and R[1:8,1] = ones(8,1).
- Feature Ranking (Fig 3B)—When |*X*| = 8>min{8−1, ⌈0.8×8⌉} = 7, *L*∪*Y* = ∅∪{9} = {9} and *X* = {1,…,8}. Then, we have, ${\left(r_{i}\right)}_{i=1}^{1}=(9)$ and ${\left(c_{j}\right)}_{j=1}^{8}=(1,\ldots ,8)$. Since p = |*L*|+n = 1 and q = |*X*| = 8, E is a 1×8 submatrix of R. When |*X*| = 5<7, *L* = {2,3,4} and *X* = {1,5,6,7,8}. Then, we have ${\left(r_{i}\right)}_{i=1}^{3}=(2,3,4)$ and ${\left(c_{j}\right)}_{j=1}^{5}=(1,5,6,7,8)$. Since p = |*L*| = 3 and q = |*X*| = 5, E is a 3×5 submatrix of R. When |*X*| = 8>7, *w*_*i*_ = 1 and *b*_*j*_ = 0. By Eq 1 and Eq 2, we calculated *d*_*w*2_ = 0.5913 and *d*_*b*2_ = 0.4087. By Eq 3, we have *s*_2_ = 0.5913. When |*X*| = 5<7, *w*_*i*_ = 1 and *b*_*i*_ = 0. By Eq 1 and Eq 2, we calculated *d*_*w*6_ = 1.1871 and *d*_*b*6_ = 0.9912. By Eq 3, we got *s*_6_ = 0.5450.
- Feature Selection (Fig 3C)—When 4 features {2,5,8,4} are selected, the accuracy of EVAL1 reached a peak of 0.803. Interestingly, with less features {2,3,4}, the accuracy of EVAL1+EVAL2 can reach a higher 0.815.
- Performance Evaluation (Fig 3D)—{2,3,4} has the lowest number of features, but the highest score among multiple performance metrics. We can see that the accuracy of {2,5,8,4,7,6,3}, {2,5,8,4} and {2,3,4} are 0.74, 0.82 and 0.87, respectively. We can also see that the F1 score of {2,5,8,4,7,6,3}, {2,5,8,4} and {2,3,4} are 0.67, 0.72 and 0.78, respectively.

### Influence of dataset uncertainty on the feature selection of the MCDM algorithm

**Fig 4:**
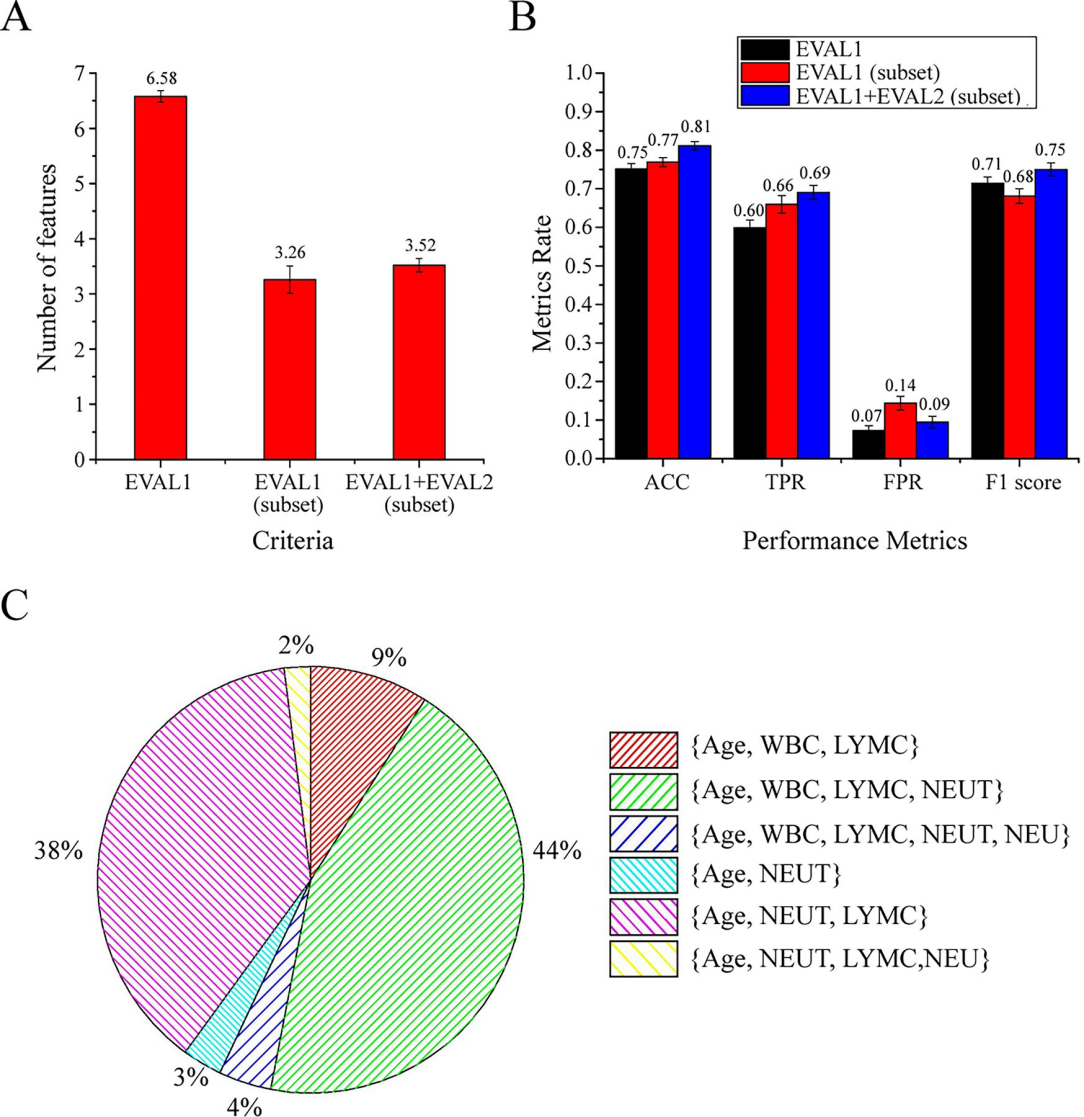
The subset of features selected by the MCDM algorithm to predict the severity of COVID-19. Data set was divided 100 times (80% training set and 20% test set) and repeatedly run the algorithm to test the stability of the algorithm and observe the influence of the dataset uncertainty on feature selection. (A). the average number of features selected by 3 different criteria. EVAL1: The correlation between input features *x*∈*X* and output features y∈*Y*, *R*[*x,y*] or *R*[*y,x*]; EVAL2: The correlation between input features *x*∈*X* and labeled features v∈*L*, *R*[*x,v*] or *R*[*v,x*]; Subset: The optimal input feature subset. Error bars represents 95% CI. (B). The metrics (ACC, TPR, FPR and F1 score) of 3 different criteria. Error bars represents 95% CI. (C). Different feature selection rates of EVAL1+ EVAL2 subsets.

To test the stability of the algorithm and observe the influence of the dataset uncertainty on feature selection, we divided the data set 100 times (80% training set and 20% test set) and repeatedly run the algorithm. The average number of features selected by 3 different criteria, EVAL1, EVAL1 (subset) and EVAL1+EVAL2 (subset) are 6.58 (95% CI: 6.48–6.68), 3.26 (95% CI: 3.01–3.51) and 3.52 (95% CI: 3.40–3.64), respectively (Fig 4A). The criteria, EVAL1+EVAL2 (subset), adopted by the MCDM algorithm improved most performance metrics. The metrics (ACC, TPR, FPR and F1 score) of EVAL1+EVAL2 (subset) are 0.81 (95% CI: 0.80–0.82), 0.69 (95% CI: 0.67–0.71), 0.09 (95% CI: 0.08–0.11) and 0.75 (95% CI: 0.73–0.77) respectively, while those of EVAL1 are 0.75 (95% CI: 0.74–0.77), 0.60 (95% CI: 0.58–0.62), 0.07 (95% CI: 0.06–0.09) and 0.71(95% CI: 0.70–0.73) respectively (Fig 4B). Although dataset uncertainties have an influence on feature selection, there were still 3 subsets: {Age, WBC, LYMC, NEUT} with a selection rate of 44%, {Age, NEUT, LYMC} with a selection rate of 38%, and {Age, WBC, LYMC} with a selection rate of 9%, which dominated EVAL1+EVAL2 (subset) feature selection. These 3 subsets can achieve high accuracy with a small number of features (Fig 4C).

### Predictive value of the features selected by the MCDM algorithm

**Fig 5:**
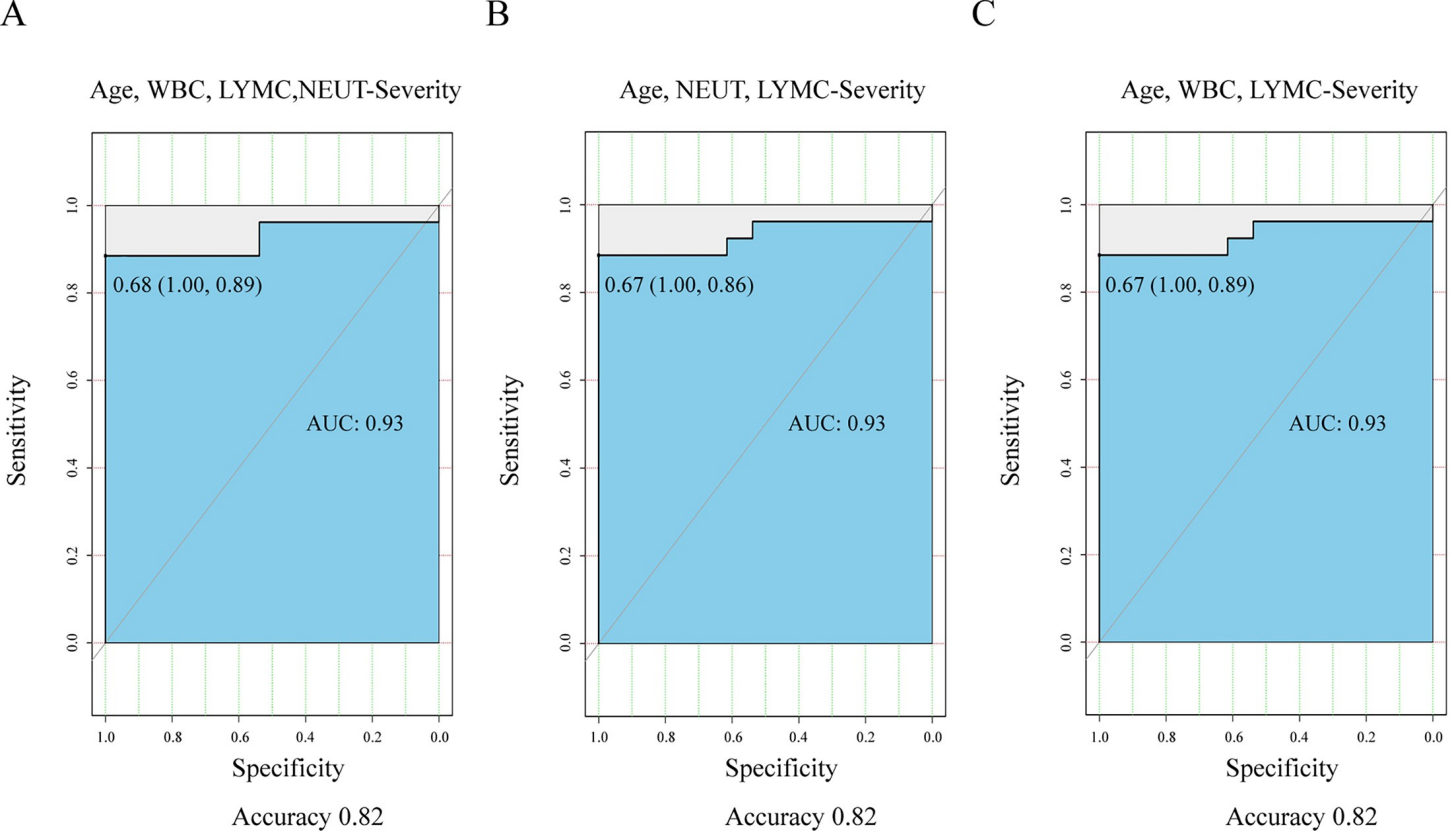
ROC curve used to evaluate the predictive value of the features selected by the MCDM algorithm for the severity of COVID-19. (A). ROC curve used to evaluate the predictive value of {Age, WBC, LYMC, NEUT} for the severity of COVID-19. (B). ROC curve used to evaluate the predictive value of {Age, NEUT, LYMC} for the severity of COVID-19. (C). ROC curve used to evaluate the predictive value of {Age, WBC, LYMC} for the severity of COVID-19. Stratified random sampling: 80% for the “training set” and 20% for the “testing set”.

Using stratified random sampling, we generated ROC curves to evaluate the predictive values of the subsets selected by the MCDM algorithm: 80% for the “training set” and 20% for the “testing set”. Our analysis results showed that {Age, WBC, LYMC, NEUT} (Fig 5A), {Age, NEUT, LYMC} (Fig 5B) and {Age, WBC, LYMC} (Fig 5C) all achieved 0.82 accuracy and 0.93 AUC. The MCDM algorithm can steadily and accurately select Age and other features from initial blood routine test results to predict the severity of COVID-19.

## Discussion

In this paper, we determined that age was the most critical indicator for predicting the severity of COVID-19. To improve the prediction accuracy, we proposed an MCDM algorithm, which combines the TOPSIS and NB classifier, to further select the indicators of patients’ initial blood routine test. By ranking features, the MCDM algorithm selected three subsets, including {Age, WBC, LYMC, NEUT}, {AGE WBC, LMYC} and {Age, NEUT, LYMC}, all of which can achieve 0.82 accuracies and 0.93 AUC.

Previous studies have shown that elderly COVID-19 patients with multiple concomitant diseases tend to develop Multiple Organ Failure (MOFE), leading to high mortality in elderly patients infected by SARS-CoV-2 [7, 10]. According to the latest meta-analysis of the elderly in the European community, the prevalence of frailty is around 15% for the elderly 65 years and older [35], and the case fatality rate of patients over 85 years old is 1,000 times that of patients aged 5–17 years [36]. Our research indicated that age was the most important indicator for predicting the severity of COVID-19, with an accuracy 0.77 and an AUC of 0.92. However, some elderly patients had a good prognosis, so prognostic evaluation and medical decision-making based on age alone might not be accurate enough.

We found that WBC, LYMC and NEUT in initial blood routine test results other than age are also crucial for predicting the severity of COVID-19. Guo et al. [37] pointed out that the MuLBSTA score revealed that multi-lobar infiltrates, lymphocytes ≤0.8×10^9^/L, bacterial infection, smoking status, hypertension, and age ≥60 years could help prognosticate outcomes in COVID-19 patients [38]. The elevated WBC/NEUT is an essential sign of bacterial infection. Bacterial co-infection in COVID-19 patients may develop a severe form of disease, complicating the clinical situation [39–41]. The control and elimination of viruses depend on humoral immunity. Viral infections usually lead to abnormal changes in lymphocyte subsets which further impaired immune system functionality. The decrease of LYMC is the most straightforward and most intuitive indicator to predict the humoral immune response, indicating that the patient’s T cell function is defective [18, 42, 43]. The count of lymphocyte subsets (CD4+ and CD8+ T cell), especially CD8+ T cell, is directly proportional to the severity of COVID-19 [44, 45].

Although logistic regression can determine the key indicator Age, and discrete analysis can find a better subset {Age, WBC LYMC}, it is difficult to determine the best subset due to the small sample size or multicollinearity. Previous studies used the MCDM algorithm to evaluate diagnostic tests [46] and help doctors hasten COVID-19 treatment [47]. As far as we know, this is the first time the MCDM algorithm has been used to predict the severity of COVID-19. It first uses TOPSIS for feature ranking, and then combines the NB classifier for feature selection. Even if the sample size is small, the MCDM algorithm can select 3 effective subsets {Age, WBC, LYMC}, {Age, WBC, LYMC, NEUT} and {Age, NEUT, LYMC}. The selection process is visual and interpretable helping doctors find the features of the progress of emerging infectious diseases early, to make faster and better prevention and treatment plans. We used the ROC curve to evaluate the predictive value of the features selected by the MCDM algorithm. The results showed that the MCDM algorithm can not only find all effective subsets, but also predict stably and accurately.

Some recent studies point out that age [48–51], underlying diseases [17], systemic immune status [52], and blood test results can be used as key features to predict the severity of COVID-19. Although these features can improve the prediction accuracy (84%~93%), the tests are time-consuming, expensive, and labor-intensive. Our algorithm can select features from blood test results to achieve a prediction accuracy of 82%. During the COVID-19 pandemic, it is more in line with clinical needs and is easy to promote and use in areas with different medical levels.

Our research provides a possible and convenient strategy for the early prediction of COVID-19 severity. However, there are some limitations associated with it. First, there were only 196 cases, and all were from China. The sample size of the study was relatively small. We would like to collect more data and conduct multi-center evaluations. Second, the patient selection process may have been affected by referral bias because of the retrospective design. Third, the screening features are all derived from blood routine tests and are relatively simple. Other features, such as chest CT, absolute T cell count, etc., can be included during the therapy to further evaluate and predict patients’ prognosis.

## Conclusion

Our research revealed that using age and the indicators WBC/NEUT and LYMC selected by the MCDM algorithm from initial blood routine test results can effectively predict the severity of COVID-19. Advanced age, combined bacterial infections, and low immunity are the main reasons leading to the severity of COVID-19. We are considered feature selection as an MCDM problem so that the algorithm could provide a reference for clinical practice. Using the most common blood routine test, medical institutions could better determine the quarantine, hospital admission, ICU assignment of COVID-19 patients. The MCDM algorithm can be used for small sample data sets, and the prediction is accurate and stable. This study not only provided a reference for establishing a rapid response mechanism in the early stage of emerging infectious disease outbreaks but also help doctors understand the pathogenesis of new infectious disease through key indicators.

## Supporting information

S1 Fig Clean data detection. After cleaning the data, there is no missing data in the dataset.
(TIF)

S1 Table Anonymized data set. For gender, 0 represent female while 1 represent male; For Severity, 0 represent Mild-Moderate while 1 represent Severe-Critically Severe.
(CSV)

## List of abbreviations

COVID-19: Coronavirus disease 2019
MCDM: Criteria Decision Making
TOPSIS: Technique for Order of Preference by Similarity to Ideal Solution
NB: Naïve Bayes
ARDS: Acute Respiratory Distress Syndrome
MODS: Multiple Organ Dysfunction Syndrome
CRP: C-reactive protein
WBC: White Blood Cell Count
LYMC: Lymphocyte Count
LYMPH: Lymphocyte Ratio
NEUT: Neutrophil Count
NEU: Neutrophil Ratio
NLR: Neutrophil to Lymphocyte Ratio
ACC: Accuracy
TPR: True Positive Rate
FPR: False Positive Rate
ROC: Receiver Operating Characteristic
AUC: Area Under the Curve
