# Supplementary figures and images for: The selection of indicators from initial blood routine test results to improve the accuracy of early prediction of COVID-19 severity
Source: PLoS One. 2021 Jun 15;16(6):e0253329. doi: 10.1371/journal.pone.0253329 (PMC8208037; doi:10.1371/journal.pone.0253329)

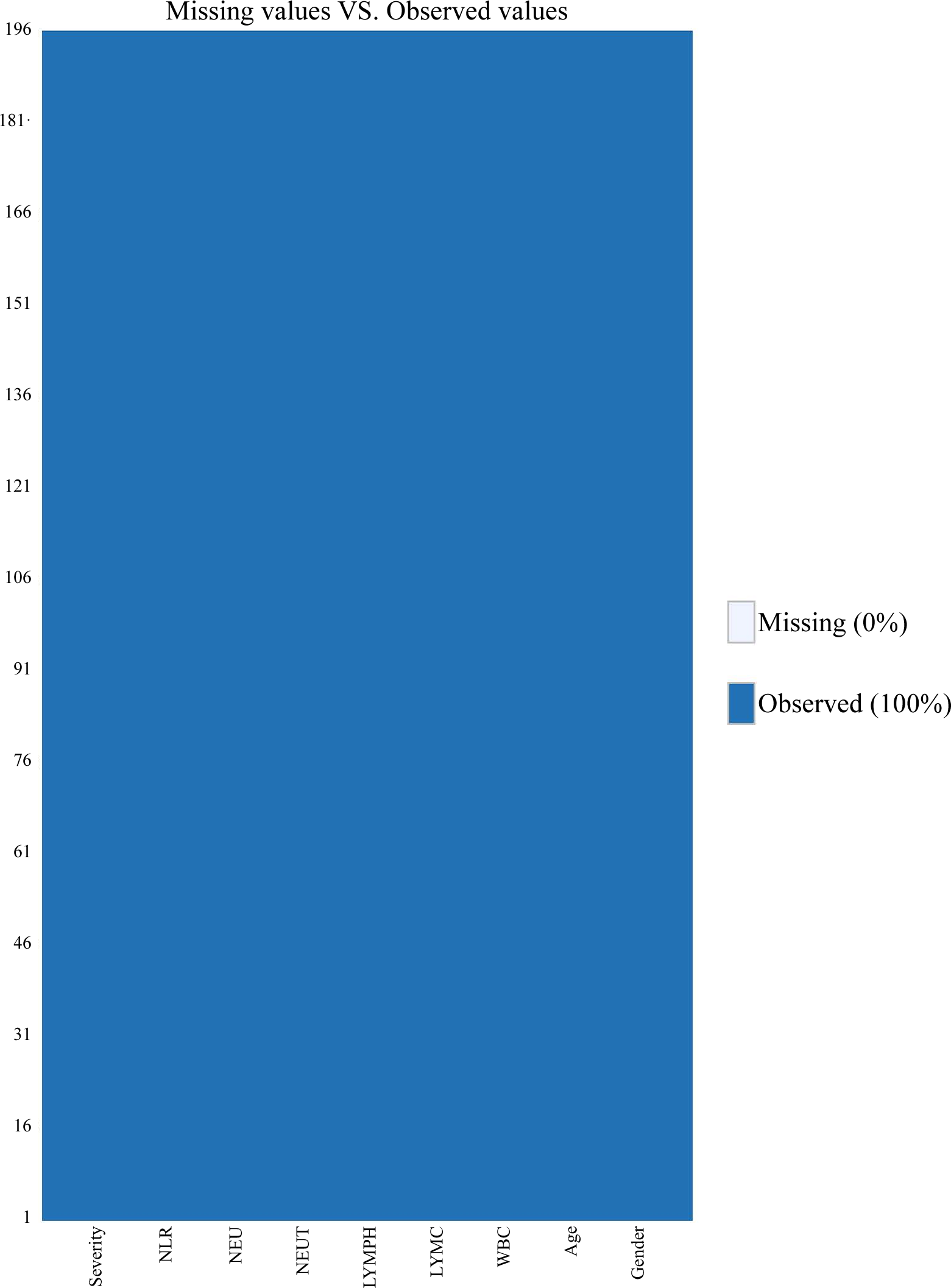

Supplement: S1 Fig — After cleaning the data, there is no missing data in the dataset. (TIF) [file pone.0253329.s001.tif]
